# Atypical Presentation of Chronic Appendicitis With Concurrent Epididymo-Orchitis in a 71-Year-Old Patient: A Case Report and Clinical Implications

**DOI:** 10.7759/cureus.94236

**Published:** 2025-10-09

**Authors:** Mark Salib, John Salib, Truc T Kha, Roman Lopez, Yoseline Escalante-Buendia, Kayla-Marie Figueroa, Frederick Tiesenga

**Affiliations:** 1 School of Medicine, St. George's University School of Medicine, St. George's, GRD; 2 General Surgery, Community First Medical Center, Chicago, USA; 3 General Surgery, West Suburban Medical Center, Oak Park, USA

**Keywords:** appendicitis, appendix, case report, chronic appendicitis, epididymo-orchitis

## Abstract

Chronic appendicitis is an uncommon and often overlooked condition that may present with recurrent, nonspecific abdominal pain, leading to significant delays in diagnosis. The clinical manifestations frequently resemble other gastrointestinal or genitourinary disorders, which further complicates recognition and timely management. We present the case of a 71-year-old man with persistent abdominal pain accompanied by testicular discomfort and swelling, an atypical presentation that obscured the underlying etiology. Through careful evaluation and imaging, the diagnosis of chronic appendicitis was established, and definitive surgical management resulted in complete resolution of symptoms. This case underscores the importance of considering chronic appendicitis in patients with persistent abdominal complaints, highlights the diagnostic value of maintaining a broad differential, and reinforces the role of surgical resection in preventing progression and associated complications.

## Introduction

Acute appendicitis is the most common surgical emergency worldwide [1], most frequently resulting from luminal obstruction, lymphatic hyperplasia, or, less commonly, neoplasms. While the pathophysiology of acute appendicitis is well established, chronic appendicitis remains a controversial and poorly understood entity. This condition presents distinct diagnostic and therapeutic challenges, as patients often display atypical or nonspecific symptoms that can easily be misattributed to other gastrointestinal or genitourinary disorders [2].

Chronic appendicitis accounts for approximately 1.5% of all appendicitis cases [3]. Its low incidence, combined with subtle and intermittent clinical manifestations, frequently leads to delayed or missed diagnoses. Although not typically considered a surgical emergency, untreated chronic appendicitis may progress to significant complications. Advanced imaging, particularly computed tomography (CT), has become invaluable in supporting diagnosis and guiding timely intervention [2].

Several predictive scoring systems have been developed to aid in the diagnosis of acute appendicitis, with the Alvarado score being among the most widely used [4]. The Alvarado score incorporates eight clinical and laboratory parameters, including migratory right lower quadrant pain, anorexia, nausea or vomiting, tenderness in the right lower quadrant, rebound tenderness, elevated temperature, leukocytosis, and a shift to the left in neutrophil count [4]. While highly useful in acute presentations, the Alvarado score is less reliable in chronic appendicitis due to the disease's indolent nature and intermittent symptomatology. Nevertheless, it provides a framework for structured assessment and underscores the importance of combining clinical judgment with imaging and laboratory evaluation in challenging cases.

The patient's presentation is notable for concurrent chronic appendicitis and epididymo-orchitis, an uncommon but clinically significant overlap. Chronic inflammation of the appendix can lead to intermittent right lower quadrant pain, which may radiate to the groin and mimic genitourinary pathology. Conversely, inflammatory processes of the epididymis and testis can produce referred pain to the lower abdomen, potentially obscuring the underlying source. In this case, the coexistence of both conditions underscores the diagnostic complexity of right lower quadrant pain. It highlights the importance of maintaining a broad differential diagnosis when evaluating chronic or recurrent abdominal and scrotal symptoms. The inflammatory cross-talk between adjacent pelvic structures may contribute to symptom persistence and overlapping clinical findings.

Thus, we present the case of a 71-year-old man with a prolonged history of recurrent abdominal pain, accompanied by testicular discomfort and swelling, which had intermittently affected his daily activities over several weeks. This case highlights the importance of maintaining a high index of clinical suspicion, integrating diagnostic imaging with thorough clinical evaluation, and recognizing when surgical intervention is necessary to prevent recurrence and potential complications.

## Case presentation

A 71-year-old man presented to the emergency department with a three-day history of persistent abdominal pain localized to the right lower quadrant and right suprapubic region, accompanied by right testicular discomfort and swelling. He also reported increased urinary frequency but denied dysuria, hematuria, or changes in urinary stream. He further denied systemic symptoms, including fever, chills, nausea, vomiting, shortness of breath, chest pain, palpitations, or cough, and had no history of recent trauma, gastrointestinal bleeding, or changes in bowel habits. His past medical history was notable for coronary artery disease, hyperlipidemia, prior myocardial infarction, and a right inguinal hernia repair approximately 24 years ago. He had no known drug allergies and was compliant with medications for cardiovascular risk management. He noted that these symptoms were atypical compared with any previous abdominal discomfort, had been progressively interfering with his daily activities, and had not prompted prior medical evaluation.

Upon examination, the patient was an elderly man who appeared fatigued and in mild distress. His vital signs were stable. Abdominal assessment revealed a soft, nondistended abdomen with localized tenderness to both superficial and deep palpation in the right lower quadrant and right suprapubic region. There was no guarding, rebound tenderness, or rigidity, and McBurney’s sign was negative. Bowel sounds were present and normoactive throughout. Genitourinary examination revealed swelling, erythema, and firmness of the right testicle without fluctuance, induration, or overlying skin changes suggestive of infection or abscess formation. The remainder of the systemic examination, including cardiovascular, respiratory, and neurological assessments, was unremarkable.

Laboratory evaluation on admission (as depicted in Table 1) demonstrated a leukocytosis of 15.9 × 10³/µL with a marked neutrophilia of 82%, consistent with an acute inflammatory response. All other parameters within the complete blood count, including hemoglobin, hematocrit, platelets, and red blood cell indices, were within normal limits, suggesting preserved hematologic function. The comprehensive metabolic panel and liver function tests were unremarkable, indicating normal renal and hepatic function. Inflammatory markers, including C-reactive protein and erythrocyte sedimentation rate, were within normal limits, reflecting a relatively localized inflammatory process rather than a systemic response. Collectively, these findings support the presence of a localized acute inflammatory condition, correlating with the patient's clinical presentation of right lower quadrant abdominal pain and right testicular discomfort.

**Table 1:** Laboratory results on admission Comprehensive laboratory evaluation, including CBC, CBC Diff, CMP, LFTs, and inflammatory markers, was done CBC, complete blood count; CBC Diff, complete blood count differential; CMP, comprehensive metabolic panel; LFTs, liver function tests; BUN, blood urea nitrogen

Category	Test	Result	Reference range
CBC	WBC	15.9 × 10³/µL	4.0-11.0 × 10³/µL
	Hemoglobin	14.2 g/dL	13.5-17.5 g/dL
	Hematocrit	42%	41-53%
	Platelets	220 × 10³/µL	150-400 × 10³/µL
	Mean corpuscular volume	90 fL	80-100 fL
	Mean corpuscular hemoglobin	30 pg	27-33 pg
	Mean corpuscular hemoglobin concentration	34 g/dL	32-36 g/dL
	Red cell distribution width	13.50%	11.5-14.5%
CBC Diff	Neutrophils	82%	40-70%
	Lymphocytes	12%	20-45%
	Monocytes	4%	2-8%
	Eosinophils	1%	1-4%
	Basophils	1%	0-1%
	Bands	2%	0-5%
	Immature granulocytes	1%	0-2%
CMP	Sodium	139 mmol/L	135-145 mmol/L
	Potassium	4.1 mmol/L	3.5-5.0 mmol/L
	Chloride	102 mmol/L	98-107 mmol/L
	Bicarbonate	25 mmol/L	22-29 mmol/L
	BUN	16 mg/dL	7-20 mg/dL
	Creatinine	1.0 mg/dL	0.7-1.3 mg/dL
	Glucose	92 mg/dL	70-100 mg/dL
LFTs	Aspartate aminotransferase	28 U/L	10-40 U/L
	Alanine aminotransferase	32 U/L	7-56 U/L
	Alkaline phosphatase	78 U/L	40-129 U/L
	Total bilirubin	0.8 mg/dL	0.1-1.2 mg/dL
	Albumin	4.3 g/dL	3.5-5.0 g/dL
Inflammatory markers	C-reactive protein	9 mg/L	<10 mg/L
	Erythrocyte sedimentation rate	15 mm/hour	0-20 mm/hour

**Figure 1:**
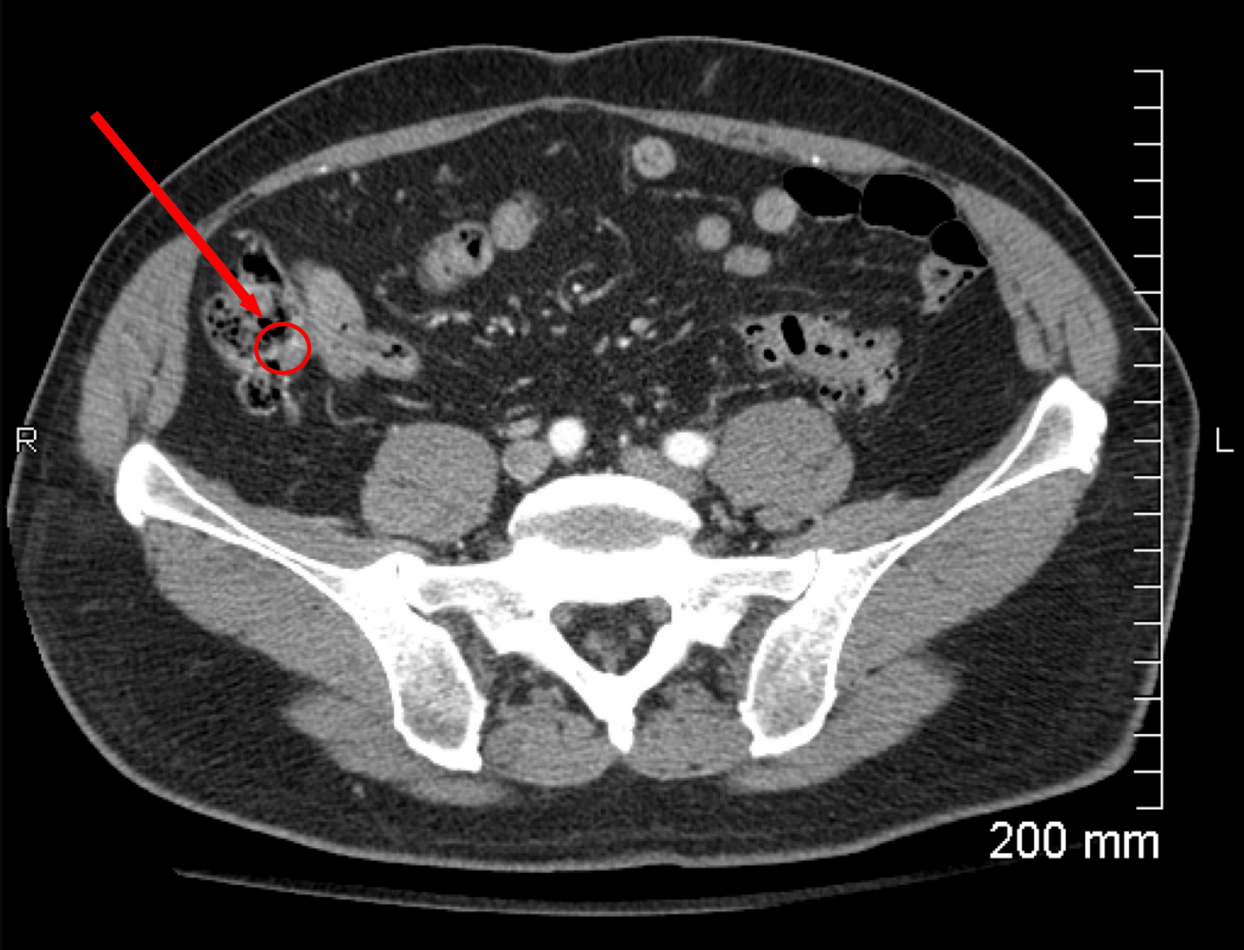
Contrast-enhanced CT of the abdomen and pelvis Axial CT demonstrates a mildly dilated appendix measuring 13 mm with periappendiceal fat stranding. The area of interest is denoted by the red arrow, consistent with chronic appendiceal inflammation CT, computed tomography

**Figure 2:**
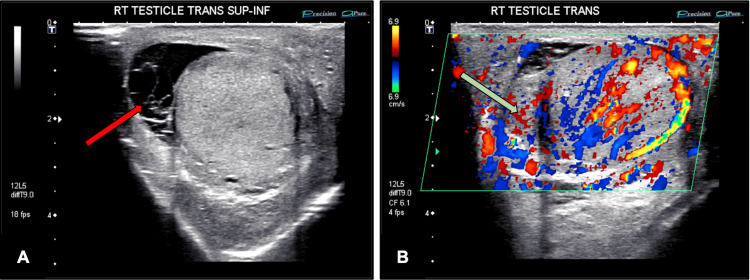
Scrotal ultrasound with and without Doppler (A) Grayscale ultrasound of the right testis demonstrates a complex, lobular moderate hydrocele (as depicted by the red arrow). (B) Color Doppler imaging reveals diffuse thickening of the right epididymal head (as depicted by the green arrow) with increased vascularity, consistent with epididymo-orchitis RT, right testicle; SUP-INF, superior-to-inferior

To further investigate the patient's abdominal pain, a contrast-enhanced CT scan of the abdomen and pelvis (Figure 1) was obtained. Imaging demonstrated a mildly dilated and thickened appendix measuring 13 mm, accompanied by periappendiceal fat stranding. When interpreted in the context of the patient's clinical presentation and laboratory findings, these results were highly suggestive of chronic appendicitis. A scrotal ultrasound was performed, both with and without Doppler (as depicted in Figure 2), revealing a complex moderate lobular hydrocele. The right epididymal head was diffusely thickened and demonstrated increased vascularity. These findings were consistent with diffuse right epididymo-orchitis in the setting of a complex moderate hydrocele, corroborating the patient’s reported right testicular pain and swelling.

Upon admission, the patient was initiated on intravenous ceftriaxone for the management of diffuse right epididymo-orchitis, along with supportive care, including cold compresses to reduce scrotal inflammation. After a thorough clinical evaluation and review of the patient's imaging and laboratory findings, the decision was made to proceed with a diagnostic laparoscopy to further assess the abdominal pathology and guide definitive management. Intraoperatively, no inguinal hernias were identified; however, multiple dense adhesions were noted surrounding the right colon, involving the omentum, abdominal wall, and cecum. The appendix was observed to be atrophic, prompting the surgical team to perform lysis of adhesions and an appendectomy to address the chronic appendicitis, and a surgical sample was sent for pathological evaluation. The procedure was completed without intraoperative complications, and hemostasis was confirmed prior to closure.

**Figure 3:**
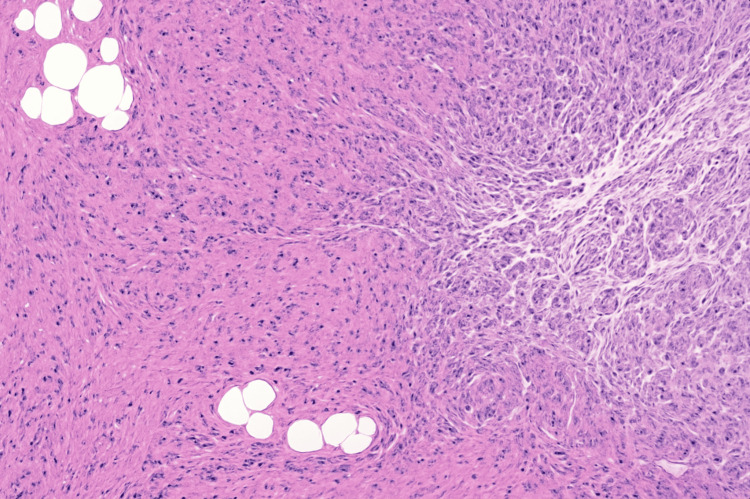
Hematoxylin and eosin-stained section of resected appendix (10× magnification) Gross examination revealed a 6.8-cm appendix with intact serosa, smooth mucosa, and mild hemorrhage. Microscopy demonstrated chronic inflammatory changes in the wall, consistent with chronic appendicitis, without neoplasia or obstruction

Tissue samples from the resected appendix were submitted for pathological analysis. Examination (Figure 3) revealed a pink-tan appendix measuring 6.8 cm in length and 0.3-0.5 cm in diameter, with an attached mesoappendix. The wall was up to 0.1 cm thick, and the serosal surface was smooth and intact, without perforations or masses. On sectioning, the lumen contained tan fluid, and the mucosa appeared smooth with mild hemorrhage. No neoplastic lesions or obstructive pathology were identified. These findings are consistent with chronic inflammatory changes, corroborating the clinical diagnosis of chronic appendicitis.

The patient was admitted for a total of four days and was discharged on postoperative day 3 after demonstrating steady clinical improvement, remaining hemodynamically stable, tolerating oral intake without difficulty, and ambulating independently. At outpatient follow-up visits over the subsequent six months, he reported complete resolution of his recurrent abdominal pain and right testicular discomfort. There were no postoperative complications, and he successfully returned to his normal daily activities within three weeks of surgery.

## Discussion

Chronic appendicitis is a rare clinical entity, representing approximately 1.5% of all appendicitis cases [5-7]. Unlike the abrupt and severe presentation of acute appendicitis, chronic appendicitis is characterized by recurrent, mild to moderate abdominal pain that may persist for weeks or months [8,9]. The underlying pathophysiology is believed to involve partial or transient obstruction of the appendiceal lumen, which can be influenced by adhesions, fibrotic scarring, fecaliths, tumors, lymphoid hyperplasia, foreign bodies, or anatomical variations such as appendiceal folding [10-12]. Histopathological examination in chronic appendicitis typically demonstrates transmural chronic inflammation without necrosis, as observed in this patient, confirming the diagnosis and explaining the insidious clinical course [13]. Such subtle presentations, especially in older adults, frequently lack classic signs such as rebound tenderness, guarding, or marked leukocytosis, complicating timely diagnosis [14].

**Figure 4:**
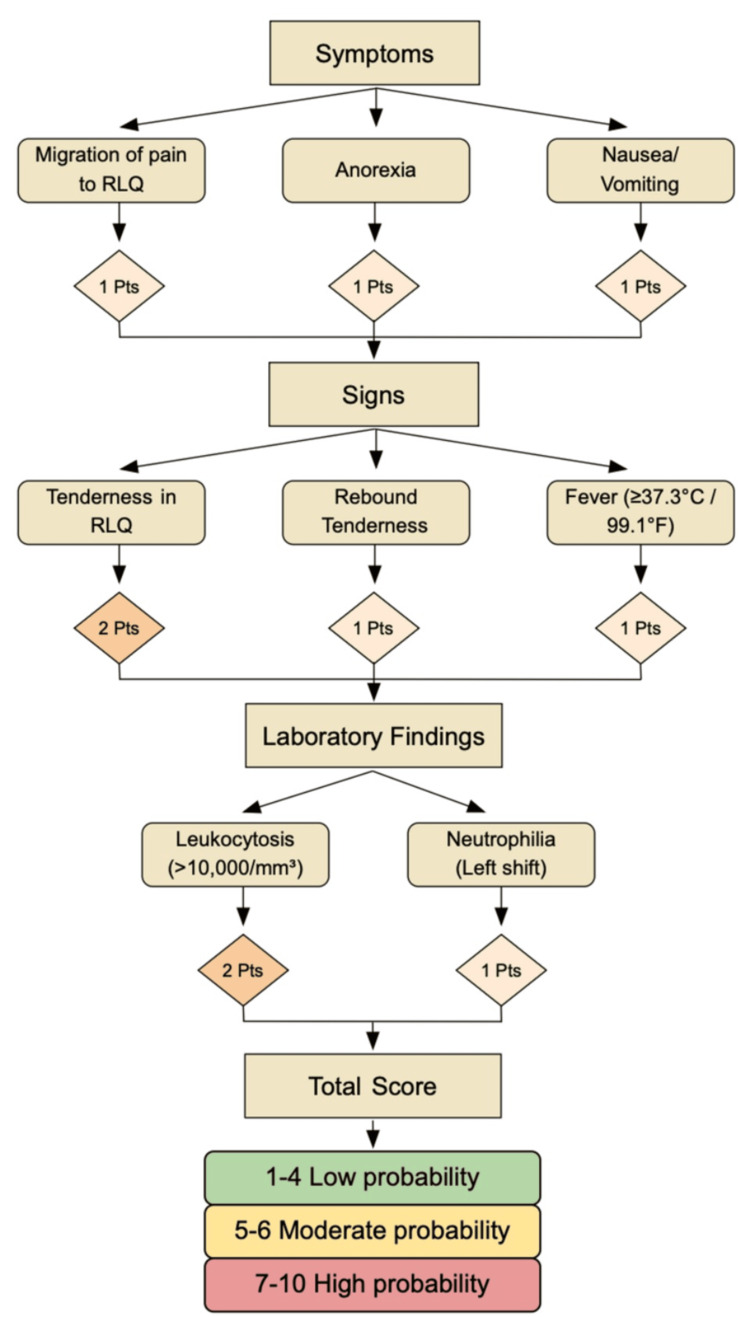
Alvarado score for the diagnosis of acute appendicitis Components of the Alvarado score (0-10 points), grouped into symptoms, signs, and laboratory findings. Risk stratification: low (0-4), intermediate (5-6), and high (7-10) [16] RLQ, right lower quadrant

Advanced imaging modalities, particularly contrast-enhanced CT, are invaluable in evaluating suspected chronic appendicitis. Typical findings may include mild appendiceal thickening, luminal dilation, or periappendiceal fat stranding, although these features can be subtle and easily overlooked [15]. In this patient, CT demonstrated a mildly thickened appendix measuring 13 mm, which, alongside clinical assessment, raised a strong suspicion for chronic appendicitis. Predictive scoring systems, such as the Alvarado score (as depicted in Figure 4), are widely used for acute appendicitis but have limited applicability in chronic presentations due to atypical symptomatology [16]. In this case, the patient's Alvarado score was 4/10, reflecting a low probability for acute appendicitis, thereby underscoring the need for a nuanced, patient-specific diagnostic strategy that integrates imaging, clinical judgment, and surgical evaluation [16].

The differential diagnosis in this patient was particularly complex, given overlapping symptoms of epididymo-orchitis and a history of prior inguinal hernia repair. Epididymo-orchitis, which is most commonly infectious in etiology in older men (e.g., *Escherichia coli*), typically presents with unilateral testicular pain, swelling, and hypervascularity on ultrasound, while noninfectious causes, including trauma, autoimmune disease, drug effects, or urine reflux, may also contribute [17,18]. Inguinal hernia recurrence was a relevant consideration given the patient's prior repair, as these often present with groin and testicular discomfort [5,6]. In this patient, scrotal ultrasound revealed a complex hydrocele with diffuse epididymal thickening and hypervascularity, consistent with epididymo-orchitis, while CT imaging highlighted appendiceal thickening without acute surrounding inflammation. These findings exemplify the diagnostic challenges of distinguishing between coexisting pathologies in older adults presenting with nonspecific abdominal and genitourinary symptoms [6,7].

Despite initial antibiotic therapy with intravenous ceftriaxone, the patient's symptoms persisted, prompting consideration for surgical exploration. Diagnostic laparoscopy was performed, revealing no recurrent hernia but dense adhesions around the right colon and an atrophic appendix. Lysis of adhesions and appendectomy were performed, definitively addressing chronic appendiceal inflammation and mechanical obstruction. This approach not only confirmed the diagnosis but also mitigated the risk of serious complications, such as perforation or abscess formation [14]. Surgical management was favored over continued conservative therapy due to the persistence of symptoms, the patient’s age, and the multifactorial etiology of his pain [15]. Postoperative recovery was uneventful, with complete resolution of both abdominal and testicular symptoms, aligning with expected outcomes for patients undergoing laparoscopic appendectomy for chronic appendicitis [12,13].

This case underscores several critical points. First, chronic appendicitis should be considered in the differential diagnosis of recurrent or atypical right lower quadrant pain, particularly in older adults with comorbid conditions or prior abdominal surgeries [5,8]. Second, reliance on predictive scoring systems alone may be insufficient, as demonstrated by the patient's low Alvarado score, necessitating a multimodal diagnostic approach that integrates imaging, clinical evaluation, and, when appropriate, surgical exploration [16,17]. Finally, this case highlights the importance of individualized management strategies, where surgical intervention remains the definitive treatment for chronic appendicitis and can safely resolve symptoms while preventing complications [14,15].

## Conclusions

This case illustrates the complexities of diagnosing and managing patients with concurrent abdominal and genitourinary conditions, particularly in older adults with atypical presentations. The patient presented with diffuse epididymo-orchitis accompanied by a hydrocele, as well as chronic appendicitis with dense adhesions. Management comprised targeted intravenous antibiotic therapy, laparoscopic appendectomy, and adhesiolysis, leading to complete resolution of symptoms. This case highlights the critical importance of maintaining a broad differential diagnosis, integrating thorough clinical assessment with imaging and laboratory findings, and exercising careful clinical judgment when evaluating multifactorial presentations. Timely and appropriately tailored surgical intervention can effectively address overlapping pathologies, prevent complications, and optimize patient outcomes.
